# My Impulsivity

**DOI:** 10.1093/schizbullopen/sgae014

**Published:** 2024-05-31

**Authors:** Jason Jepson

I often wonder why I feel the need to post on social media when my post will sometimes be considered insignificant. Is it just the instant gratification of seeing if people agree with me? How productive is it really when I share my story online repeatedly?

I googled my mental illness recently and learned something new about my diagnosis of schizoaffective disorder. I learned that impulsiveness can be a characteristic of schizophrenia. I quickly recognized impulsivity as a problem in my social media posts. Like many, I want instant gratification from others when I share my thoughts about mental illness online. Even reading about impulsivity, I felt the immediate need to post about my own impulsivity.

In the past, I had been warned by my editor about impulsive posting, but I just shrugged it off, thinking I knew better. Recently I have been thinking about posting the minute an idea pops into my head, but I need to take the time to think clearly about every post. Will it help my reader? Is it accurate? Do I really have to talk about my schizophrenia every day? Am I being putting myself on display as a mentally ill person, and not as a schizophrenia advocate, which I am proud of being? We are not all famous, but I often incorrectly assume my last online post will give me the credibility I desire. My validation comes from the number of “likes” I see after my posts.

As a man living with schizophrenia, I have to understand my schizophrenia and do my best to be more productive off-screen. I can use my impulsivity to be positive in my writing. Going forward, I want to be firm with myself about posting impulsively. I can go outside for a walk, cultivating the thought before I post it online. Even a good night’s sleep can change whether or not I decide to post my thoughts about my mental illness.

People like to be heard. Three things I try to convey in my advocacy are that schizophrenia can be treated, recovery is possible, and you are not alone. For me, I do not know how I feel about being out there for the world to see, at least for now. I need a good night’s sleep to think about it. Maybe I will post this thought tomorrow, or maybe not.

